# Supplementary material for: Tooth discoloration caused by nanographene oxide as an irrigant and intracanal medicament in the endodontic treatment of extracted single-rooted teeth: An ex-vivo study
Source: PLoS One. 2025 Jun 26;20(6):e0325430. doi: 10.1371/journal.pone.0325430 (PMC12200680; doi:10.1371/journal.pone.0325430)
Supplement: S2 Table — *T1 (immediately after being placed inside the canal.), **T2 (one week later), ***T3 (one month later), and ****T4 (three months later). Having the same uppercase English letters in each vertical column indicates no statistically significant differences between the groups (p < 0.05). (DOCX) [file pone.0325430.s002.docx]

**Table 2. The mean and standard deviation of the discolorations of the intracanal medicament.**

*T1 (immediately after being placed inside the canal.), **T2 (one week later), ***T3 (one month later), and ****T4 (three months later). Having the same uppercase English letters in each vertical column indicates no statistically significant differences between the groups (p<0.05).
