# Supplementary material for: Tooth discoloration caused by nanographene oxide as an irrigant and intracanal medicament in the endodontic treatment of extracted single-rooted teeth: An ex-vivo study
Source: PLoS One. 2025 Jun 26;20(6):e0325430. doi: 10.1371/journal.pone.0325430 (PMC12200680; doi:10.1371/journal.pone.0325430)
Supplement: Attachment — Submitted filename: PONE-D-24-30140_reviewer.pdf [file pone.0325430.s003.pdf]

PLOS ONE

Tooth discoloration caused by nanographene oxide as an irrigant and intracanal medicament in the endodontic treatment of extracted single-rooted teeth: an ex-vivo study

--Manuscript Draft--

**Manuscript Number:**	PONE-D-24-30140
**Article Type:**	Research Article
**Full Title:**	Tooth discoloration caused by nanographene oxide as an irrigant and intracanal medicament in the endodontic treatment of extracted single-rooted teeth: an ex-vivo study
**Short Title:**	Discoloration by nanographene oxide in Endodontics
**Corresponding Author:**	Zeinab Rafiee<br>Shiraz University of Medical Sciences Faculty of Dentistry<br>Shiraz, IRAN, ISLAMIC REPUBLIC OF
**Keywords:**	Tooth discoloration; Nanographene oxide; Intracanal medicament; Root canal Irrigant
**Abstract:**	Aim<br>Tooth discoloration resulting from intracanal medicaments and irrigants poses aesthetic challenges in dentistry. The aim of this study was to investigate the discoloration of teeth caused by nanoparticles and to compare it with that caused by other common materials used in endodontic treatment.<br>Materials and Methods<br>One hundred and eight single-rooted, single-canal anterior mandibular and maxillary premolar teeth, extracted due to orthodontic treatment or periodontal disease, were selected and prepared. The specimens were randomly divided into seven experimental groups and two control groups, each consisting of 12 samples. The experimental groups included three irrigant groups: nanographene oxide, sodium hypochlorite, and positively charged imidazolium silver nanoparticles. The four medicament groups were nanographene oxide-carboxymethyl cellulose, imidazolium coated silver nanoparticles-carboxymethyl cellulose, calcium hydroxide, and carboxymethyl cellulose. The control groups were normal saline and blood. Discoloration was examined at five time points: before the materials were placed (T0), immediately after placement (T1), one week later (T2), one month later (T3), and three months later (T4). Discoloration was measured using spectrophotometry, and the resulting ΔE values were analyzed statistically using ANOVA tests with repeated measurements and Tukey's post-hoc ANOVA.<br>Results<br>After three months, there was no statistically significant difference in discoloration among the irrigants ($P>0.05$). The highest degree of discoloration among the irrigants was observed in the imidazolium coated silver nanoparticles group. The ΔE values in the normal saline group (negative control) showed significant differences compared to the imidazolium ($p=0.001$) and blood ($p=0.007$) groups. Among the intracanal medicaments, a significant difference in ΔE values was found between the carboxymethyl cellulose and calcium hydroxide groups ($p=0.005$) at the final examination (three months). There were no significant differences among the other groups ($P>0.05$).<br>Conclusion<br>Nanographene oxide, as both an irrigant and medicament, does not cause more discoloration than other commonly used materials in endodontic treatment. Therefore, it can be considered a viable alternative to traditional materials.
**Order of Authors:**	Abbas Abbaszadegan, DDS, MSc
	Zeinab Rafiee
	Bahar Asheghi
	Ahmad Gholami
**Additional Information:**	

Powered by Editorial Manager® and ProduXion Manager® from Aries Systems Corporation

Question	Response
**Financial Disclosure**<br><br>Enter a financial disclosure statement that describes the sources of funding for the work included in this submission. Review the submission guidelines for detailed requirements. View published research articles from *PLOS ONE* for specific examples.<br><br>This statement is required for submission and **will appear in the published article** if the submission is accepted. Please make sure it is accurate.<br><br>**Funded studies**<br>Enter a statement with the following details:<br>• Initials of the authors who received each award<br>• Grant numbers awarded to each author<br>• The full name of each funder<br>• URL of each funder website<br>• Did the sponsors or funders play any role in the study design, data collection and analysis, decision to publish, or preparation of the manuscript?<br><br>Did you receive funding for this work?	Yes
Please add funding details.<br>as follow-up to "**Financial Disclosure**<br><br>Enter a financial disclosure statement that describes the sources of funding for the work included in this submission. Review the submission guidelines for detailed requirements. View published research articles from *PLOS ONE* for specific examples.<br><br>This statement is required for submission and **will appear in the published article** if the submission is accepted. Please make sure it is accurate.	The Vice-Chancellery of Shiraz University of Medical Sciences for supporting this study (Grant #24674)

**Funded studies**<br>Enter a statement with the following details:<br>• Initials of the authors who received each award<br>• Grant numbers awarded to each author<br>• The full name of each funder<br>• URL of each funder website<br>• Did the sponsors or funders play any role in the study design, data collection and analysis, decision to publish, or preparation of the manuscript?<br><br>Did you receive funding for this work?"	
Please select the country of your main research funder (please select carefully as in some cases this is used in fee calculation).<br>as follow-up to "**Financial Disclosure**<br><br>Enter a financial disclosure statement that describes the sources of funding for the work included in this submission. Review the submission guidelines for detailed requirements. View published research articles from *PLOS ONE* for specific examples.<br><br>This statement is required for submission and **will appear in the published article** if the submission is accepted. Please make sure it is accurate.<br><br>**Funded studies**<br>Enter a statement with the following details:<br>• Initials of the authors who received each award<br>• Grant numbers awarded to each author<br>• The full name of each funder<br>• URL of each funder website<br>• Did the sponsors or funders play any role in the study design, data collection and analysis, decision to publish, or preparation of the manuscript?<br><br>Did you receive funding for this work?"	IRAN, ISLAMIC REPUBLIC OF - IR
**Competing Interests**	NO authors have competing interests

Use the instructions below to enter a competing interest statement for this submission. On behalf of all authors, disclose any competing interests that could be perceived to bias this work—acknowledging all financial support and any other relevant financial or non-financial competing interests.<br><br>This statement is required for submission and **will appear in the published article** if the submission is accepted. Please make sure it is accurate and that any funding sources listed in your Funding Information later in the submission form are also declared in your Financial Disclosure statement.<br><br>View published research articles from *PLOS ONE* for specific examples.<br><br>**NO authors have competing interests**<br><br>Enter: *The authors have declared that no competing interests exist.*<br><br>**Authors with competing interests**<br><br>Enter competing interest details beginning with this statement:<br><br>*I have read the journal's policy and the authors of this manuscript have the following competing interests: [insert competing interests here]*<br><br>* typeset	
**Ethics Statement**<br><br>Enter an ethics statement for this submission. This statement is required if the study involved:<br><br>• Human participants<br>• Human specimens or tissue<br>• Vertebrate animals or cephalopods<br>• Vertebrate embryos or tissues	This study received approval from the ethics committee of Shiraz University of Medical Sciences (IR.SUMS.DENTAL.REC.1400.120).

Powered by Editorial Manager® and ProduXion Manager® from Aries Systems Corporation

• Field research<br><br>Write "N/A" if the submission does not require an ethics statement.<br><br>General guidance is provided below. Consult the submission guidelines for detailed instructions. **Make sure that all information entered here is included in the Methods section of the manuscript.**	

**Format for specific study types**<br><br>**Human Subject Research (involving human participants and/or tissue)**<br>• Give the name of the institutional review board or ethics committee that approved the study<br>• Include the approval number and/or a statement indicating approval of this research<br>• Indicate the form of consent obtained (written/oral) or the reason that consent was not obtained (e.g. the data were analyzed anonymously)<br><br>**Animal Research (involving vertebrate animals, embryos or tissues)**<br>• Provide the name of the Institutional Animal Care and Use Committee (IACUC) or other relevant ethics board that reviewed the study protocol, and indicate whether they approved this research or granted a formal waiver of ethical approval<br>• Include an approval number if one was obtained<br>• If the study involved *non-human primates*, add *additional details* about animal welfare and steps taken to ameliorate suffering<br>• If anesthesia, euthanasia, or any kind of animal sacrifice is part of the study, include briefly which substances and/or methods were applied<br><br>**Field Research**<br><br>Include the following details if this study involves the collection of plant, animal, or other materials from a natural setting:<br>• Field permit number<br>• Name of the institution or relevant body that granted permission	
**Data Availability**<br><br>Authors are required to make all data underlying the findings described fully available, without restriction, and from the time of publication. PLOS allows rare exceptions to address legal and ethical concerns. See the PLOS Data Policy and FAQ for detailed information.	Yes - all data are fully available without restriction

A Data Availability Statement describing where the data can be found is required at submission. Your answers to this question constitute the Data Availability Statement and **will be published in the article**, if accepted.<br><br>**Important:** Stating 'data available on request from the author' is not sufficient. If your data are only available upon request, select 'No' for the first question and explain your exceptional situation in the text box.<br><br>Do the authors confirm that all data underlying the findings described in their manuscript are fully available without restriction?	
**Describe where the data may be found in full sentences. If you are copying our sample text, replace any instances of XXX with the appropriate details.**<br><br>• If the data are **held or will be held in a public repository**, include URLs, accession numbers or DOIs. If this information will only be available after acceptance, indicate this by ticking the box below. For example: *All XXX files are available from the XXX database (accession number(s) XXX, XXX.*).<br>• If the data are all contained **within the manuscript and/or Supporting Information files**, enter the following: *All relevant data are within the manuscript and its Supporting Information files.*<br>• If neither of these applies but you are able to provide **details of access elsewhere**, with or without limitations, please do so. For example:<br><br>*Data cannot be shared publicly because of [XXX]. Data are available from the XXX Institutional Data Access / Ethics Committee (contact via XXX) for researchers who meet the criteria for access to confidential data.*<br><br>*The data underlying the results presented in the study are available from (include the name of the third party*	The data underlying the results presented in the study are available.

and contact information or URL).<br>• This text is appropriate if the data are owned by a third party and authors do not have permission to share the data.<br><br>* typeset	
Additional data availability information:	

Powered by Editorial Manager® and ProduXion Manager® from Aries Systems Corporation

Manuscript.doc Click here to access/download;Manuscript;manuscript (1).docx

Tooth discoloration caused by nanographene oxide as an irrigant and intracanal medicament in the endodontic treatment of extracted single-rooted teeth: an ex-vivo study

Running title: Tooth discoloration and nanographene oxide

Abbas Abbaszadegan[1] Zeinab Rafiee*[2] Bahar Asheghi*[3] Ahmad Gholami[4]

[1] Associated professor, Dept. of Endodontics, School of Dentistry, Shiraz University of Medical Sciences, Shiraz, Iran.

ORCID ID: 0000-0003-4637-3550 Email: dr.abbaszadegan@gmail.com

Phone: +989173028781

[2]Post graduate student, Dept. of Endodontics, School of Dentistry, Shiraz University of Medical Sciences, Shiraz, Iran.

ORCID ID: 000-0001-6712-9982 Email: Nobakhtf78@gmail.com

Phone: +989131616641

[3]Assistant professor, Dept. of Endodontics, School of Dentistry, Shiraz University of Medical Sciences, Shiraz, Iran.

ORCID ID: 0000-0001-6798-7549 Email: Bahar64a@gmail.com

Phone:+989382923249

[4] Associated professor, Biotechnology Research Center, School of Pharmacy, Shiraz University of Medical Sciences, Shiraz, Iran.

ORCID ID: 0000-0003-1851-159X Email: ahmadgholami60@gmail.com

Phone: +989173140518.

Main Correspondent: Zeinab Rafiee, Post graduate student, Dept. of Endodontics, School of Dentistry, Shiraz University of Medical Sciences, Shiraz, Iran.Email:nobakhtf78@gmail.com Phone: +989131616641

Co-correspondent: Bahar Asheghi, Assistant professor, Dept. of Endodontics, School of Dentistry, Shiraz University of Medical Sciences, Shiraz, Iran. Email: Bahar64a@gmail.com, Phone: +989173140518

Tooth discoloration caused by nanographene oxide as an irrigant and intracanal medicament in the endodontic treatment of extracted single-rooted teeth: an ex-vivo study

Aim

Tooth discoloration resulting from intracanal medicaments and irrigants poses aesthetic challenges in dentistry. The aim of this study was to investigate the discoloration of teeth caused by nanoparticles and to compare it with that caused by other common materials used in endodontic treatment.

Materials and Methods

One hundred and eight single-rooted, single-canal anterior mandibular and maxillary premolar teeth, extracted due to orthodontic treatment or periodontal disease, were selected and prepared. The specimens were randomly divided into seven experimental groups and two control groups, each consisting of 12 samples. The experimental groups included three irrigant groups: nanographene oxide, sodium hypochlorite, and positively charged imidazolium silver nanoparticles. The four medicament groups were nanographene oxide-carboxymethyl cellulose, imidazolium coated silver nanoparticles-carboxymethyl cellulose, calcium hydroxide, and carboxymethyl cellulose. The control groups were normal saline and blood. Discoloration was examined at five time points: before the materials were placed (T0), immediately after placement (T1), one week later (T2), one month later (T3), and three months later (T4). Discoloration was measured using spectrophotometry, and the resulting ΔE values were analyzed statistically using ANOVA tests with repeated measurements and Tukey's post-hoc ANOVA.

Results

After three months, there was no statistically significant difference in discoloration among the irrigants ($P>0.05$). The highest degree of discoloration among the irrigants was observed in the imidazolium coated silver nanoparticles group. The ΔE values in the normal saline group (negative control) showed significant differences compared to the imidazolium ($p=0.001$) and blood ($p=0.007$) groups. Among the intracanal medicaments, a significant difference in ΔE values was found between the carboxymethyl cellulose and calcium hydroxide groups ($p=0.005$) at the final examination (three months). There were no significant differences among the other groups ($P>0.05$).

Conclusion

Nanographene oxide, as both an irrigant and medicament, does not cause more discoloration than other commonly used materials in endodontic treatment. Therefore, it can be considered a viable alternative to traditional materials.

Keywords: tooth discoloration, nanographene oxide, intracanal medicament, irrigant

Introduction

Reducing the microbial load in the root canal system is the aim of endodontic treatment (1). Removing the bacterial biofilm from the root canal system completely is not possible solely through mechanical preparation. Hence, intracanal medicaments and irrigants are employed in the root canal system to disinfect the inaccessible areas (2).

The most common intracanal medicaments are triple antibiotic paste and calcium hydroxide ($Ca(OH)_2$), whereas the most common irrigants are sodium hypochlorite and chlorhexidine (CHX) (3). The disadvantages of common materials such as cytotoxicity, change in the structure of dentin, questionable disinfection ability, and tooth discoloration have led studies toward removing these limitations in order to obtain ideal materials.

Dr. Freitas Jr. introduced the field of nanotechnology in dentistry in 2000 under the title of ‘nano-dentistry’ (4). Nanoparticles (with a diameter of ≤100 nm) have become popular antimicrobial agents due to their broad antibacterial activity as well as anti-biofilm and biocompatibility effects. Their larger charge density, surface area, and area lead to more interaction with bacteria. Recent studies have paid attention to the use of nanoparticles in endodontic treatment due to their chemical and physical characteristics (5).

Although nanoparticles are effective for the disinfection of the root canal, their potential negative effects such as the discoloration of teeth make them questionable for in vivo use, notably for long-term applications as irrigants and intracanal medicaments (6-8).

Due to the unique morphology of carbon-based nanoparticles, researches have shifted towards carbon-based nanomaterials. Nanographene oxide is a major chemical derivative of graphene and is more biocompatible than it.

As one of the most important chemical derivatives of graphene, nanographene oxide easily connects with polymers, biomolecules, DNAs, and proteins with the presence of a functional group on its surface. In addition, it has been shown that, due to its hydrophilicity, it is more biocompatible than graphene which is hydrophobic (9). Since graphene is a valuable material for studies, the problems in its clinical use such as possible tooth discoloration should be investigated.

Only a few studies have investigated the discoloration caused by nanographene oxide. In one study, the researchers investigated the tooth discoloration after using silver nanoparticles in combination with nanographene oxide as an intracanal medicament (3). Their results showed that the combination of silver nanoparticles with nanographene oxide caused less discoloration than the combination of calcium hydroxide and silver nanoparticles.

Considering that tooth discoloration significantly impacts smile aesthetics, it is important not to solely focus on the biological aspects of nanomaterials. On the other hand, as no study has addressed the discoloration caused by nanographene oxide alone or in combination with other medicaments as an irrigant and intracanal medicament, the goal of the current research is to investigate the discoloration induced by nanographene oxide at different time points and compare it with that caused by common medicaments used in endodontic treatment.

Materials and methods

This study received approval from the Ethics Committee of Shiraz University of Medical Sciences (IR.SUMS.DENTAL.REC.1400.120). In the present research, single-rooted and single-canal anterior mandibular and maxillary premolar teeth extracted due to orthodontic treatment or periodontal disease between June 3, 2022 and December 25, 2022 were used. These teeth were referred, for extraction, to the surgery ward of Shiraz Dental School. Written informed consent was obtained from the patients.

Preparation of the teeth

One hundred eight single-rooted teeth with straight roots, without caries, without crown fracture, with mature apex, and without cervical abrasion were considered in the present research. The teeth were examined for cracks and resorption using a microscope (Zeiss, Oberkochen, Germany). The surface debris and calculus were polished using a scaler (Supprasson, Satelec, France), a pumice paste, and a rubber cap. To ascertain that the teeth had a single canal, periapical radiographs were taken from two angles. In order to standardize the length of the tooth roots, 2-4 mm of their ends was sectioned. The root canals of the teeth were prepared from the apical side to the most coronal part of the pulp chamber. Each tooth was prepared using a ProTaper rotary file (Dentsply Maillefer, Ballaigues, Switzerland) based on the instructions of the manufacturer (S1, S2, F1, F2, and F3, respectively). Then, the preparation of the teeth was

completed using Gates Gidden drills 1 to 6 (Mani, Tochigi, Japan). During the preparation, 5 mL of 2.5% sodium hypochlorite was utilized between the instrumentations. To eliminate any residual pulp tissue at the end of root canal preparation, 5.5% sodium hypochlorite mechanically activated using a #3 ultrasonic tip (ProUltra, Dentsply, Johnson City, TN, USA) connected to an ultrasonicator (Supprasson; Satelec, France) was used.

To eliminate the smear layer and expose the dentin tubules, 17% EDTA (Aria Dent Tehran, Iran) was used to wash the canals for two minutes. After drying the canal with paper cones, 5 mL of the tested material was introduced into the canal for 30 minutes and replaced during this time period. Finally, using a 27-gauge syringe, the canals were washed with 5 mL of normal saline. The apical end of the canal was sealed with a restorative glass (Fuji Corporation, Tokyo, Japan). During the test, the samples were kept in boxes with 100% humidity within an incubator at a temperature of 37 °C. The teeth were mounted with silicone molding materials so that possible displacement could be prevented.

Preparation of the irrigants

The teeth were put into three groups (each consisting of 12 samples) at random according to the irrigant employed during the preparation of the canal. Blood was considered the positive control group, whereas normal saline was considered the negative control group. According to the study of Eskandari et al., nanographene oxide was synthesized and characterized using Hummers' method (10). Moreover, for the synthesis of silver nanoparticles with imidazolium coating, the method in the study of Gholami et al. was used (11).

Preparing the teeth for placing the intracanal medicament

Based on the inclusion criteria, forty-eight teeth similar to those in the irrigant group were selected. The teeth were prepared from the coronal region to ensure the placement of medicaments below the CEJ of the standard access cavities by employing a fissure bur (d&z, Wiesbaden, Germany) and a round bur (Dentsply Maillefer, Ballaigues, Switzerland). Then, they were kept in physiological saline until use. Afterward, similar to the preparation method for the irrigant, the canal was prepared. Lastly, 5 mL of normal saline was used as the final irrigant to wash the root canals of the teeth. After drying the canal with a paper cone, a sufficient and equal amount of the desired paste was placed to the area below the CEJ using a Lentulo spiral. Then,

the teeth were sealed with restorative glass. The samples were put into four groups of 12 each at random. The first group was CaOH2 (Golchai Dent, Iran), the second group was AgNp+-CMC (Merck, Germany), the third group was GONps-CMC, and the fourth group was CMC (Afa Chemi, Tehran, Iran). It should be noted that for the preparation of medicaments containing nanographene oxide and silver-imidazolium nanoparticles, each of these materials was mixed with carboxymethyl cellulose.

Evaluation of tooth discoloration

The VITA Easyshade Compact (Vita Zahnfabrik, Bad Säckingen, Germany) spectrophotometer was used for measuring the degree of tooth discoloration considering the accuracy and reproducibility of the measurement. The discolorations were examined at 5 stages: T0 (before the injection of materials), T1 (immediately after the injection of materials), T2 (one week later), T3 (one month later), and T4 (three months later).

The measurements were performed by a technician in a room with the same light in an area 3 mm above the CEJ of the buccal surface of the tooth. We repeated the measurements three times for each tooth and recorded their mean. The Commission International de l'Eclairage (CIE) L*a*b* color system was employed to report the data. The discolorations between two measurements were computed according to the equation below:

$\Delta E^* = [(L_1 - L_0^*)+(a_1 - a_0^*)+(b_1 - b_0^*)^2]$ ½

Statistical analyses

To compare and statistically analyze the discolorations after using different materials, the Kolmogorov-Smirnov test was first utilized to evaluate the normality of the distribution of the color difference data. Tukey’s post-hoc and the ANOVA tests as well as the statistical significance level of 0.05 were used for comparison as well as finding the causes of differences. The IBM SPSS Statistics 26 software was used for statistical analysis.

Results

All the irrigants used in this study caused discoloration immediately after being placed inside the canal. This discoloration continued for three months (Fig. 1).

According to the obtained ΔE values, all the materials exhibited discoloration over the three-month period. Normal saline and AgNp+ exhibited the least and most discoloration, respectively. There was a significant difference in the ΔE values between the normal saline group and the AgNp+ ($p=0.00$) and blood ($p=0.00$) groups. There was no significant difference between the other groups ($P>0.05$). The discolorations at different time periods are shown in Table 1.

It should be noted that the intracanal medicaments used in this study caused discoloration immediately after being placed inside the canal. This discoloration continued for three months (Fig. 2).

According to the obtained ΔE values, all the materials exhibited discoloration over the three-month period. CMC showed the least discoloration, while $CaOH_2$ demonstrated the most discoloration. There was a significant difference in ΔE values between the CMC and $CaOH_2$ groups ($p=0.00$). There was no significant difference between the other groups ($p>0.05$). Table 2 shows the discolorations at different time periods.

Discussion

One of the issues associated with root canal-treated teeth is discoloration resulting from materials used in endodontic procedures which can impact the aesthetics of the tooth (12). As such, new materials used in endodontic treatment should also be aesthetically pleasing in addition to possessing properties such as antibacterial effects and biocompatibility.

This study is the first to investigate the discoloration caused by nanographene oxide as an irrigant and intracanal medicament over time compared to other common endodontic treatment materials. The obtained results showed that the discoloration caused by nanographene oxide was similar to those of other common materials used in endodontic treatment and that the time and method of its use (as irrigant or intracanal medicament) do not affect the discoloration.

Similar to other studies, in this study, the spectrophotometer method was used to investigate and evaluate tooth discoloration (13, 14) because spectrophotometers are very accurate tools that can

show minor discolorations that are not clinically visible. In addition, they provide more reliable results for evaluating tooth discoloration than other methods (15).

To simulate the clinical conditions more closely, in the present study, similar to other studies, extracted human teeth were used to evaluate the discoloration caused by different materials because these teeth exhibit similar color patterns to natural teeth in the oral cavity (13). In order to create a closed system and maintain the seal of the samples for the group of irrigants, the teeth were prepared and cleaned from their apex. In the intracanal medicament groups, access was made from the tooth crown to ensure the placement of the medicaments below the CEJ. This method was selected and applied similar to previous studies (13, 16-18). In addition, blood was used as the positive control group because it can undoubtedly change the color of teeth (12, 19, 20).

Furthermore, in this study, the smear layer was removed to enhance the penetration of the medicaments into the dentin tubules. This step was necessary because the smear layer can affect dentin permeability and alter tooth color, potentially leading to prolonged discoloration (16).

In this study, carboxymethyl cellulose (CMC) was used as a carrier for nanographene oxide and silver nanoparticles coated with imidazolium because this carrier can make the transport and distribution of intracanal medicaments easier. Moreover, it can improve the physical and chemical interactions between the intracanal medicament and the inner wall of the dentin (21).

As one of the most important chemical derivatives of graphene, nanographene oxide (GO) readily binds with polymers, biomolecules, DNA, and proteins since there are functional groups on its surface. Additionally, its hydrophilicity renders it more biocompatible compared to graphene which is hydrophobic. Nanographene oxide exhibits strong antimicrobial properties and high regenerative capacity. Therefore, in this study, nanographene oxide was utilized instead of nanographene. However, potential issues such as tooth discoloration should also be investigated for its clinical application (22). Our study showed that as an irrigant and intracanal medicament, this substance does not lead to a significant discoloration compared to normal saline and other tested substances. It is noteworthy that this substance led to tooth discoloration in all time intervals. However, there were no significant differences in the degree of discoloration between different time intervals. Moreover, the results indicated that all the irrigants employed in this study led to discoloration in different time intervals.

In this study, we investigated silver nanoparticles coated with imidazolium, previously recognized for their antimicrobial properties, to assess their potential for inducing tooth discoloration. Our findings revealed significant discoloration from this substance after one month compared to normal saline, with these changes persisting from one to three months. This suggests that silver nanoparticle materials may increase discoloration over time possibly due to the gradual substitution of tooth calcium by the metal ions present in the compound.

Consistent with our results, Moazami et al. (17) also noted significant and progressive discoloration associated with silver nanoparticles coated with imidazolium as irrigants, compared to other materials, across one-to-three-month intervals in their study.

The results of comparing intracanal medicaments in this study revealed that most of the tested materials exhibited progressively increasing discoloration at various intervals up to three months. Among them, calcium hydroxide demonstrated more pronounced changes compared to the other materials. While the disparity between calcium hydroxide and other substances was significant within the first month of use, by the three-month mark, its differences were only notable compared to carboxymethyl cellulose.

In a research performed by Betancourt et al. (23), it was witnessed that 28 days following the application of calcium hydroxide as an intracanal medicament, the resulting discoloration was less pronounced compared to other medicaments investigated such as doxycycline and clindamycin. The variance between the findings of this study and ours may be attributed to differences in the materials under investigation.

In a separate study, Zare Jahromi et al. (18) investigated tooth discoloration following the application of triple antibiotic paste and calcium hydroxide as intracanal medicaments, assessing the discoloration levels up to four months post-placement. Their findings indicated that discoloration increased significantly in both groups over time compared to baseline. Nonetheless, the difference in discoloration was not statistically significant between the two groups. Similar to our findings, the study noted significant discoloration associated with the use of calcium hydroxide.

Our study revealed that discoloration resulting from silver nanoparticles coated with imidazolium and nanographene oxide progressed over three months. However, within the first

month, the discoloration induced by calcium hydroxide was significantly greater than that caused by silver nanoparticles coated with imidazolium and nanographene oxide. By the end of three months, all three substances exhibited similar levels of discoloration, with no significant differences observed.

In a research performed by Hernández-Sierra et al. (24), the authors investigated the discoloration induced by silver nanoparticles applied as a paste on enamel surfaces. Their findings demonstrated that the discoloration resulting from this substance was significantly lower compared to those of other materials. This outcome contrasts with the results of our study which could be attributed to variations in nanoparticle synthesis methods and their different application forms.

In the study of Jaju et al. (3) conducted in 2021, the authors investigated the discoloration caused by a compound consisting of silver nanoparticles and nanographene oxide used as an intracanal medicament. Their findings revealed that this compound induced less discoloration compared to the combination of silver nanoparticles and calcium hydroxide over a one-month period. Notably, since the individual effects of nanographene oxide and silver nanoparticles were not explored separately in their research, their results cannot be directly compared to those of the present study.

In conclusion, given the nearly equivalent discoloration potential of nanographene oxide compared to other substances studied, alongside its notable biological and biocompatible properties derived from carbon, further investigations into its additional characteristics and properties concerning dentin are suggested. Moreover, it is recommended that more studies be undertaken to explore various bleaching methods and their efficacy, enabling greater confidence in the clinical application of these substances in the future.

Conclusion

Despite the limitations of the current research, it was shown that the discoloration induced by nanographene oxide was similar to those of other common substances used in endodontic treatment and the time and method of its use did not influence the degree of discoloration.

Acknowledgment

This manuscript is based on the postgraduate thesis by Dr. Zeinab Rafiee. The authors would like to express their appreciation to the Vice-Chancellery of Shiraz University of Medical Sciences for supporting this study (Grant #24674). We also thank Dr. Alireza Nobakht for the English language editing of this paper and Dr. Ahmad Baseri for his assistance with statistical analyses.

References

1. DaSilva L, Finer Y, Friedman S, Basrani B, Kishen A. Biofilm formation within the interface of bovine root dentin treated with conjugated chitosan and sealer containing chitosan nanoparticles. J Endod. 2013 Feb;39(2):249-53.

2. Afkhami F, Elahy S, Mahmoudi-Nahavandi A. Spectrophotometric analysis of crown discoloration following the use of silver nanoparticles combined with calcium hydroxide as intracanal medicament. J Clin Exp Dent. 2017 Jul 1;9(7):e842-e847

3. Jaju K,Nasim I. Evaluation Of Tooth Discolouration Following The Use Of Silver Nanoparticle Based Intracanal Medicaments - An In Vitro Study. Int J Dentistry Oral Sci. 2021;8(6):3214-3218. 32

4. Raura N, Garg A, Arora A, Roma M. Nanoparticle technology and its implications in endodontics: a review. Biomater Res. 2020 Dec 4;24(1):21.
5. Shrestha A, Shi Z, Neoh KG, Kishen A. Nanoparticulates for antibiofilm treatment and effect of aging on its antibacterial activity. J Endod. 2010 Jun;36(6):1030-5.
6. Afkhami F, Pourhashemi SJ, Sadegh M, Salehi Y, Fard MJ. Antibiofilm efficacy of silver nanoparticles as a vehicle for calcium hydroxide medicament against Enterococcus faecalis. J Dent. 2015 Dec;43(12):1573-9.
7. Javidi M, Afkhami F, Zarei M, Ghazvini K, Rajabi O. Efficacy of a combined nanoparticulate/calcium hydroxide root canal medication on elimination of Enterococcus faecalis. Aust Endod J. 2014 Aug;40(2):61-5.
8. Wu D, Fan W, Kishen A, Gutmann JL, Fan B. Evaluation of the antibacterial efficacy of silver nanoparticles against Enterococcus faecalis biofilm. J Endod. 2014 Feb;40(2):285-90.
9. Ge Z, Yang L, Xiao F, Wu Y, Yu T, Chen J, Lin J, Zhang Y. Graphene Family Nanomaterials: Properties and Potential Applications in Dentistry. Int J Biomater. 2018 Dec 9;2018:1539678.
10. Eskandari F, Ghahramani Y, Abbaszadegan A, Gholami A. The antimicrobial efficacy of nanographene oxide and double antibiotic paste per se and in combination: part II. BMC Oral Health. 2023 May 2;23(1):253.
11. Gholami A, Ghezelbash K, Asheghi B, Abbaszadegan A, Amini A. An in vitro study on the antibacterial effects of chlorhexidine-loaded positively charged silver nanoparticles on enterococcus faecalis. J Nanomater. 2022;Available in https://doi.org/10.1155/2022/6405772.
12. Lenherr P, Allgayer N, Weiger R, Filippi A, Attin T, Krastl G. Tooth discoloration induced by endodontic materials: a laboratory study. Int Endod J. 2012 Oct;45(10):942-9.
13. Afkhami F, Elahy S, Nahavandi AM, Kharazifard MJ, Sooratgar A. Discoloration of teeth due to different intracanal medicaments. Restor Dent Endod. 2019 Feb 12;44(1):e10.
14. Kim MA, Rosa V, Min KS. Effect of two graphene derivatives on Enterococcus faecalis biofilms and cytotoxicity. Dent Mater J. 2023 Mar 30;42(2):211-217.
15. Johnston WM, Kao EC. Assessment of appearance match by visual observation and clinical colorimetry. J Dent Res. 1989 May;68(5):819-22.

16. Adl A, Javanmardi S, Abbaszadegan A. Assessment of tooth discoloration induced by biodentine and white mineral trioxide aggregate in the presence of blood. J Conserv Dent. 2019 Mar-Apr;22(2):164-168.
17. Moazami F, Sahebi S, Ahzan S. Tooth Discoloration Induced by Imidazolium Based Silver Nanoparticles as an Intracanal Irrigant. J Dent (Shiraz). 2018 Dec;19(4):280-286.
18. Zare Jahromi M, Barati M, Barati J. Coronal Discoloration Potential after using two root canal dressings (calcium-Hydroxide vs. Triantibiotic Dressing). J Islam Dent Assoc Iran.2012;24(4):204-8.
19. Partovi M, Al-Havvaz AH, Soleimani B. In vitro computer analysis of crown discolouration from commonly used endodontic sealers. Aust Endod J. 2006 Dec;32(3):116-9.
20. Felman D, Parashos P. Coronal tooth discoloration and white mineral trioxide aggregate. J Endod. 2013 Apr;39(4):484-7.
21. Yaghmoor RB, Platt JA, Spolnik KJ, Chu TMG, Yassen GH. Effect of hydrogel-based antibiotic intracanal medicaments on crown discoloration. Restor Dent Endod. 2021 Oct 5;46(4):e52.
22. Qi X, Jiang F, Zhou M, Zhang W, Jiang X. Graphene oxide as a promising material in dentistry and tissue regeneration: A review. Smart Mater Med. 2021;2:280-91.
23. Betancourt P, Bucchi C, Arroyo-Bote S. Determination of crown discoloration and fluorescence induced by different medications used in regenerative endodontic procedures: An *ex vivo* study. J Clin Exp Dent. 2021 Aug 1;13(8):e755-e761.
24. Hernández-Sierra JF, Ruíz F, Castanedo-Cázares JP, Martinez-Ruiz V, Mandeville P, Pierdant-Pérez M, Gordillo-Moscoso A, Pozos-Guillén Ade J. In vitro determination of the chromatic effect of a silver nanoparticles solution linked to the gantrez S-97 copolymer on tooth enamel. J Clin Pediatr Dent. 2010 Fall;35(1):65-8.

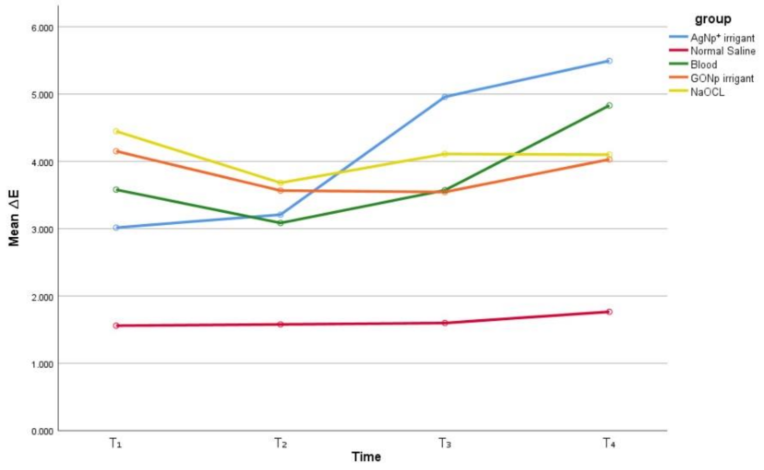

Fig. 1. The mean tooth discolorations after using canal irrigants at different time periods

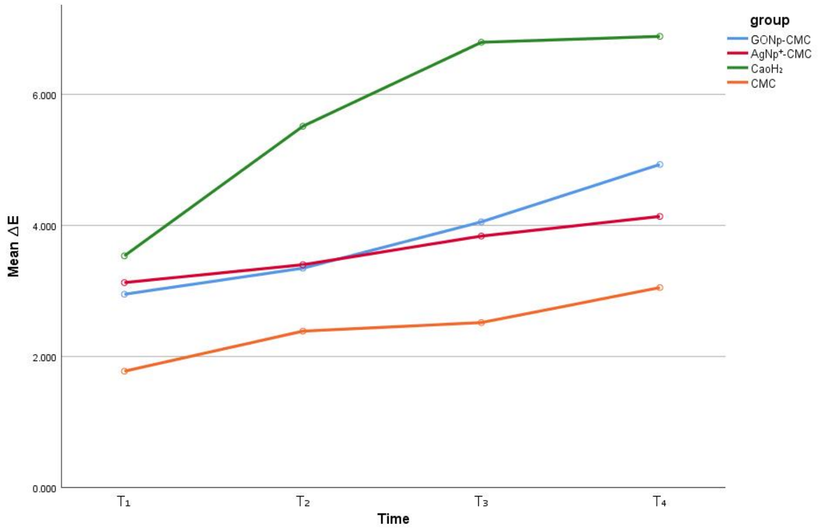

Fig. 2. The mean tooth discolorations after the use of intracanal medicaments at different time periods

Group (subgroup)	T_1	T_2	T_3	T_4
Normal saline	1.56 ± 0.54^{A}	1.58 ± 0.061^{A}	1.60 ± 0.41^{AC}	1.77 ± 0.55^{A}
$AgNp^{+}$ irrigant	3.02 ± 2.41^{AC}	3.21 ± 2.28^{AC}	4.96 ± 2.43^{BC}	5.49 ± 3.13^{BC}
GONp irrigant	4.15 ± 2.4^{BC}	3.57 ± 1.60^{AC}	3.54 ± 1.94^{AC}	4.03 ± 1.99^{AC}
NaOCL	4.45 ± 3.19^{BC}	3.68 ± 2.25^{BC}	4.11 ± 2.16^{BC}	4.10 ± 2.51^{AC}
Blood	3.58 ± 1.79^{AC}	3.08 ± 1.66^{AC}	3.57 ± 1.23^{AC}	4.83 ± 1.52^{BC}

Table 1. The mean and standard deviation of the discolorations of canal irrigants. Having the same uppercase English letters in each vertical column indicates that there are no statistically significant differences between the groups ($p<0.05$).

Group (subgroup)	T_1	T_2	T_3	T_4
GONp-CMC	2.95 ± 1.37^{A}	3.35 ± 1.89^{AC}	4.05 ± 2.24^{AC}	4.93 ± 2.57^{AC}
$AgNP^{+}$-CMC	3.13 ± 1.97^{A}	3.40 ± .1.95^{AC}	3.84 ± 2.43^{A}	4.14 ± 3.42^{AC}
$CaOH_2$	3.54 ± 2.31^{A}	5.51 ± 2.22^{B}	6.80 ± 3.26^{BC}	6.89 ± 2.86^{BC}
CMC	1.78 ± 1.03^{A}	2.39 ± 0.75^{A}	2.52 ± 1.25^{A}	3.05 ± 1.12^{A}

Table 2. The mean and standard deviation of the discolorations of the intracanal medicament. Having the same uppercase English letters in each vertical column indicates that there are no statistically significant differences between the groups ($p<0.05$).
