# Supplementary material for: Tooth discoloration caused by nanographene oxide as an irrigant and intracanal medicament in the endodontic treatment of extracted single-rooted teeth: An ex-vivo study
Source: PLoS One. 2025 Jun 26;20(6):e0325430. doi: 10.1371/journal.pone.0325430 (PMC12200680; doi:10.1371/journal.pone.0325430)
Supplement: Attachment — Submitted filename: Response to reviewers.docx [file pone.0325430.s005.docx]

**PONE-D-24-30140
Tooth discoloration caused by nanographene oxide as an irrigant and intracanal medicament in the endodontic treatment of extracted single-rooted teeth: an ex-vivo study**


**Dear Editor**,

We would like to express our sincere gratitude for your valuable comments, which have significantly helped improve the quality of our manuscript. In response, we have thoroughly revised the manuscript and addressed all the concerns raised by the reviewers in a point-by-point manner. The changes have been highlighted in the revised manuscript. Please find below our responses to the reviewers’ comments.

Thank you for your consideration.

Kind regards,
***Zeinab Rafiee***

1. PLOS ONE Style Requirements
Comment: Please ensure that your manuscript meets PLOS ONE’s style requirements, including those for file naming. The PLOS ONE style templates can be found at:
<https://journals.plos.org/plosone/s/file?id=wjVg/PLOSOne_formatting_sample_main_body.pdf> and
<https://journals.plos.org/plosone/s/file?id=ba62/PLOSOne_formatting_sample_title_authors_affiliations.pdf>

Response:
We have adjusted the reference formatting and ensured that our manuscript now adheres to all PLOS ONE style requirements, including those for file naming.

2. Grant Information Discrepancy
Comment: The grant information provided in the ‘Funding Information’ and ‘Financial Disclosure’ sections do not match. Please ensure the correct grant numbers are provided in the ‘Funding Information’ section.

Response:
The grant number and ethics approval number are distinct as per our institutional policy. To avoid confusion, we have selected and included the appropriate code in the ‘Funding Information’ section.

3. Financial Disclosure and Role of Funders
Comment: Please state the role of the funders in your study. If they had no role, include the statement: “The funders had no role in study design, data collection and analysis, decision to publish, or preparation of the manuscript.”

Response:
We confirm that the funders had no role in study design, data collection and analysis, decision to publish, or preparation of the manuscript. This statement has been added to the cover letter, and we kindly ask you to update the online submission form accordingly.

4. Funding in Acknowledgments Section
Comment: Please remove any funding-related text from the Acknowledgments section. Funding information should only appear in the Funding Statement.

Response:
We have removed the grant number and funding-related text from the manuscript body and Acknowledgments section as per your policy. The Funding Statement has been updated accordingly.

5. Competing Interests
Comment: Please declare any Competing Interests. If there are none, state: “The authors have declared that no competing interests exist.”

Response:
We have declared that no competing interests exist, and this information has been provided in the cover letter. Please update the online submission form accordingly.

6. Data Availability Statement
Comment: Please provide a complete Data Availability Statement in the submission form.

Response:
We have provided a complete Data Availability Statement in the submission form, stating: “All data are included in the manuscript and/or supporting information files.”

**Reviewer Comments:

Reviewer #1:**

1. Sample Size Calculation
Comment: Please add a sample size calculation in the Materials and Methods section with relevant references.

Response:
The sample size used in this study was based on a previous calculation performed using G*Power 3.1 software, and this has been added to the Materials and Methods section.

2. Results Clarification
Comment: Please explain the Results section in more detail.

Response:
We have expanded the Results section to provide greater clarity.

3. Tables Enhancement
Comment: Tables need to be more detailed, including headers for columns to improve clarity.

Response:
The tables have been revised to include headers and provide a clearer presentation of the data. Statistical significance is indicated for each group and time point.

4. Grammar and Presentation
Comment: Other comments regarding grammar and presentation have been highlighted in the manuscript.

Response:
All grammatical and presentation-related comments have been addressed and revised in the manuscript.

**Reviewer #2:**

1. Keywords Revision
Comment: Revise the keywords to include at least six MeSH terms.

Response:
The keywords have been revised as suggested, and now include six MeSH terms related to the study.

2. Introduction Update
Comment: The introduction should be updated with the latest information, including references to recent literature.

Response:
The introduction has been updated for better readability and to incorporate the suggested references. We have also included information on the sustainability of this work, which opens new avenues for future research in sustainable dental biomaterials.

3. Supporting Literature for Statements
Comment: Provide original studies to support specific statements in the Introduction.

Response:
We have cited the recommended original studies to support the statements regarding commonly used irrigants and nanoparticles, enhancing the scientific foundation of the Introduction.

4. Material Preparation and Esthetic Validation
Comment: Provide more details on the materials preparation and esthetic validation methods.

Response:
Two new subheadings have been added to the Materials and Methods section to detail the preparation of the irrigants and medicaments. Esthetic validation was performed using the VITA Easyshade Compact spectrophotometer, and this information has been included in the manuscript.

5. Results and Discussion Revision
Comment: The Results and Discussion sections need to be revised for clarity, and the linkage to previous work should be explained more clearly.

Response:
Both the Results and Discussion sections have been extensively rewritten for clarity, and connections to previous work are now better explained.

6. Conclusion Revision
Comment: The conclusion needs to be revised to better summarize the study’s findings and future prospects.

Response:
The Conclusion section has been revised to more effectively summarize the findings and highlight potential future directions in dental biomaterials research.

Thank you for considering our revised manuscript. We look forward to your feedback.

Best regards,
*Zeinab Rafiee*
