# Supplementary material for: Tooth discoloration caused by nanographene oxide as an irrigant and intracanal medicament in the endodontic treatment of extracted single-rooted teeth: An ex-vivo study
Source: PLoS One. 2025 Jun 26;20(6):e0325430. doi: 10.1371/journal.pone.0325430 (PMC12200680; doi:10.1371/journal.pone.0325430)
Supplement: Attachment — Submitted filename: Response_to_reviewer_comments_auresp_3.docx [file pone.0325430.s008.docx]

**PONE-R2-24-30140
Tooth discoloration caused by nanographene oxide as an irrigant and intracanal medicament in the endodontic treatment of extracted single-rooted teeth: an ex-vivo study**


**Dear Editor**,

We would like to express our sincere gratitude for your valuable comments, which have significantly helped improve the quality of our manuscript. In response, we have thoroughly revised the manuscript and addressed all the concerns raised by the reviewers in a point-by-point manner. The changes have been highlighted in the revised manuscript.

Thank you for your consideration.

Kind regards,
***Zeinab Rafiee***
