# Supplementary material for: Tooth discoloration caused by nanographene oxide as an irrigant and intracanal medicament in the endodontic treatment of extracted single-rooted teeth: An ex-vivo study
Source: PLoS One. 2025 Jun 26;20(6):e0325430. doi: 10.1371/journal.pone.0325430 (PMC12200680; doi:10.1371/journal.pone.0325430)
Supplement: Attachment — Submitted filename: Response to reviewer comments.docx [file pone.0325430.s007.docx]

**PONE-D-24-30140
Tooth discoloration caused by nanographene oxide as an irrigant and intracanal medicament in the endodontic treatment of extracted single-rooted teeth: an ex-vivo study**


**Dear Editor**,

We would like to express our sincere gratitude for your valuable comments, which have significantly helped improve the quality of our manuscript. In response, we have thoroughly revised the manuscript and addressed all the concerns raised by the reviewers in a point-by-point manner. The changes have been highlighted in the revised manuscript. Please find below our responses to the reviewers’ comments.

Thank you for your consideration.

Kind regards,
***Zeinab Rafiee***

**Reviewer Comments:

Reviewer #1:**

Reviewer #1: All previous comments have been addressed by the author.
1. The reference for Evaluation of Tooth discoloration needs to be added in the methodology.

Response:
The mentioned reference was added to the methodology section of the manuscript.

Thank you for considering our revised manuscript. We look forward to your feedback.

Best regards,
*Zeinab Rafiee*
